# Canadian COVID-19 Outbreak Surveillance System: implementation of national surveillance during a global pandemic

**DOI:** 10.17269/s41997-023-00766-5

**Published:** 2023-04-19

**Authors:** Erin McGill, Cameron Coulby, Demy Dam, Anna Bellos, Rachel McCormick, Kaitlin Patterson

**Affiliations:** 1 Infectious Disease Programs Branch, Public Health Agency of Canada, Ottawa, Ontario Canada; 2 Infectious Disease Programs Branch, Public Health Agency of Canada, Toronto, Ontario Canada; 3 Infectious Disease Programs Branch, Public Health Agency of Canada, Guelph, Ontario Canada; 4 Infectious Disease Programs Branch, Public Health Agency of Canada, Moncton, New Brunswick Canada

**Keywords:** COVID-19, Outbreak, Surveillance, Public health, Infectious disease, COVID-19, éclosion, surveillance, santé publique, maladie transmissible

## Abstract

**Setting:**

Early in the SARS-CoV-2 pandemic, the need to develop systematic outbreak surveillance at the national level to monitor trends in SARS-CoV-2 outbreaks was identified as a priority for the Public Health Agency of Canada (PHAC). The Canadian COVID-19 Outbreak Surveillance System (CCOSS) was established to monitor the frequency and severity of SARS-CoV-2 outbreaks across various community settings.

**Intervention:**

PHAC engaged with provincial/territorial partners in May 2020 to develop goals and key data elements for CCOSS. In January 2021, provincial/territorial partners began submitting cumulative outbreak line lists on a weekly basis.

**Outcomes:**

Eight provincial and territorial partners, representing 93% of the population, submit outbreak data on the number of cases and severity indicators (hospitalizations and deaths) for 24 outbreak settings to CCOSS. Outbreak data can be integrated with national case data to supply information on case demographics, clinical outcomes, vaccination status, and variant lineages. Data aggregated to the national level are used to conduct analyses and report on outbreak trends. Evidence from CCOSS analyses has been useful in supporting provincial/territorial outbreak investigations, informing policy recommendations, and monitoring the impact of public health measures (vaccination, closures) in specific outbreak settings.

**Implications:**

The development of a SARS-CoV-2 outbreak surveillance system complemented case-based surveillance and furthered the understanding of epidemiological trends. Further efforts are required to better understand SARS-CoV-2 outbreaks for Indigenous populations and other priority populations, as well as create linkages between genomic and epidemiological data. As SARS-CoV-2 outbreak surveillance enhanced case surveillance, outbreak surveillance should be a priority for emerging public health threats.

## Résumé

**Contexte:**

Au début de la pandémie de SRAS-CoV-2, l’Agence de la santé publique du Canada (ASPC) a déterminé comme priorité la nécessité de développer un système de surveillance systématique des éclosions à l’échelle nationale afin de suivre les tendances des éclosions de SRAS-CoV-2. Le système canadien de surveillance des éclosions de COVID-19 (SCSEC) a été établi pour surveiller la fréquence et la gravité des éclosions de SRAS-CoV-2 dans différents milieux communautaires.

**Intervention:**

L’ASPC s’est engagée avec les partenaires provinciaux et territoriaux en mai 2020 pour élaborer des objectifs et des éléments de données clés pour le SCSEC. En janvier 2021, les partenaires provinciaux et territoriaux ont commencé à transmettre des listes d’éclosions cumulatives hebdomadaires.

**Résultats:**

Huit partenaires provinciaux et territoriaux, représentant 93 % de la population, transmettent au SCSEC des données sur les éclosions sur le nombre de cas et les indicateurs de gravité (les hospitalisations et les décès) pour 24 types de milieux. Les données sur les éclosions peuvent être intégrées avec les données nationales sur les cas pour obtenir des informations sur la démographie des cas, les résultats cliniques, le statut vaccinal et les lignées de variants. Les données agrégées à l’échelle nationale sont utilisées pour effectuer des analyses et faire rapport des tendances sur les éclosions. Les résultats des analyses du SCSEC ont été utiles pour soutenir les enquêtes provinciales/territoriales sur les éclosions, informer les recommandations politiques et surveiller l’impact des mesures de santé publique (la vaccination, les fermetures) dans des milieux d’éclosions spécifiques.

**Implications:**

Le développement d’un système de surveillance des éclosions de SRAS-CoV-2 a permis de complémenter la surveillance des cas et d’approfondir notre compréhension des tendances épidémiologiques. Des efforts supplémentaires sont nécessaires pour mieux comprendre les éclosions de SRAS-CoV-2 chez les populations autochtones et d’autres populations minoritaires, ainsi que pour créer des liens entre les données génomiques et les données épidémiologiques. Comme la surveillance des éclosions de SRAS-CoV-2 a enrichi la surveillance des cas, la surveillance des éclosions devrait être une priorité pour les menaces émergentes pour la santé publique.

## Setting

The first confirmed SARS-CoV-2 infection in a Canadian was identified on January 25, 2020 (Sunnybrook Hospital, 2020). The Public Health Agency of Canada (PHAC) initialized case-based surveillance in February 2020. On March 11, 2020, the World Health Organization (WHO) declared SARS-CoV-2 a pandemic (Zhao et al., 2020). Shortly thereafter, community transmission of SARS-CoV-2 was observed in many regions across Canada (Waldner et al., 2021). SARS-CoV-2 outbreaks^1^ have been associated with many COVID-19 cases, hospitalizations, and deaths (Akhtar-Danesh et al., 2022; Zylke & Bauchner, 2020). Outbreaks can quickly result in large numbers of infections, potentially increasing community-based transmission through secondary exposures (Murti et al., 2021). Monitoring outbreak trends can provide critical information in the context of an emerging pathogen, as they can identify populations that may be disproportionally affected (e.g. long-term care residents, foreign/migrant workers, underhoused people). This can enable public health to better allocate resources for prevention and response to outbreaks in these settings (Brown et al., 2021; Ontario Hospital Association, 2022; Tsai & Wilson, 2020).

Senior leadership at PHAC identified a gap in our understanding of SARS-CoV-2; national case surveillance, although crucial, did not provide sufficient information on outbreak events to inform public health response. As a result, PHAC determined that it would be necessary to develop a separate, complementary outbreak-based surveillance system for SARS-CoV-2 in Canada in order to monitor trends in SARS-CoV-2 outbreaks and provide consistent data to inform the national SARS-CoV-2 response. The need for outbreak surveillance was also identified by provincial and territorial health authorities, some of whom publicly report outbreak trends on their public health website (Institut national de santé publique du Québec, 2022; Public Health Ontario, 2022).

Surveillance at the national level is important, as jurisdictions across Canada have varying experiences with COVID-19 outbreaks due to differences in population demographics and public health mitigation strategies. Understanding the national picture was important for decision-making and to capture the unique experiences of the provinces/territories. Few countries were able to develop and implement national COVID-19 outbreak surveillance, notable exceptions being Ireland and the United Kingdom with public reports focusing on outbreak settings, temporal trends, and severity indicators (outbreak-associated hospitalizations and deaths) (Health Protection Surveillance Centre, 2022; UK Health Security Agency, 2022).

A database of outbreak events, based on publicly available information from provincial/territorial websites, press briefings, and media articles, was created and used as an interim measure to track outbreaks to provide a national snapshot ahead of implementation of the Canadian COVID-19 Outbreak Surveillance System (CCOSS). Systematic web scraping, that is the identification and extraction of information from websites, of outbreak-related information began in March 2020 and contributed to PHAC’s situational awareness of SARS-CoV-2 outbreak trends in a timely manner for all provinces and territories. Although timely, there were challenges around the consistency and quality of the information, highlighting the need for a more formalized surveillance system of COVID-19 outbreaks.

The national SARS-CoV-2 outbreak surveillance system created was novel and differed from existing surveillance systems at PHAC. In Canada, the responsibility for delivering health services, including public health services, lies with provincial and territorial governments. As a result, federal public health surveillance systems rely on the participation of provincial and territorial governments to build a national dataset. To ensure representative and accurate national COVID-19 outbreak reporting, PHAC worked with provinces and territories to develop and implement the CCOSS.

The reporting and analysis of outbreaks as a unit of interest itself is rare in the published literature for established pathogens. The data collected from CCOSS provided the ability to produce timely outbreak trends on intensity, settings, and severity across jurisdictions.

The purpose of CCOSS was to systematically monitor the frequency and severity of outbreak events by setting, with the overarching goal of increasing the effectiveness of public health response and public health measures within high-risk settings and thereby minimizing the size, impact, and occurrence of COVID-19 outbreaks in Canada. The frequency of data submissions, the level of detail captured, and the preparation of reports were extremely ambitious given the urgent data needs of public health officials. This level of detail sets it apart from other existing national outbreak surveillance systems, which may focus on monitoring the number/intensity of outbreaks (e.g. FluWatch) or on using case-based surveillance systems to identify potential outbreaks (e.g. enteric lab-based surveillance) (Public Health Agency of Canada, 2018, 2022b).

Herein, we describe the process of developing and implementing this novel national outbreak surveillance system during a global pandemic to inform public health response, where public health resources were constrained and evidence on SARS-CoV-2 was rapidly evolving.

## Intervention

### Concept

**Fig. 1:**
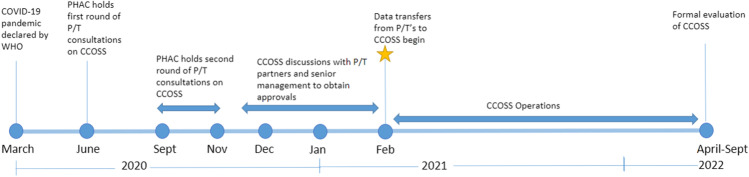
Timeline of development and implementation of the Canadian COVID-19 Outbreak Surveillance System (CCOSS) in collaboration with provincial and territorial (P/T) partners

In May 2020, PHAC engaged with provincial and territorial partners to develop the goals and key data elements for CCOSS (Fig. 1). The pandemic emergency orders were leveraged to permit rapid data sharing that pre-pandemic would have had lengthy reporting delays to PHAC. Provincial and territorial partners provided feedback on data elements of interest and on their ability to contribute to national COVID-19 outbreak surveillance.

The proposed goals of CCOSS identified in June 2020 were to:

1. Determine when and where outbreaks are occurring, and which populations are affected
2. Monitor outbreak trends by setting and population
3. Assess risk factors associated with outbreaks
4. Investigate the relative impact of outbreaks on the national burden of COVID-19
5. Evaluate outbreak control strategies
6. Describe enhanced epidemiological factors associated with COVID-19 outbreaks

### Data collection and linkage to case data

During the provincial and territorial consultations, essential data required to analyze national outbreak trends and optional variables were identified for CCOSS submission. The data elements that provinces and territories agreed to share included administrative variables (e.g. date outbreak declared, outbreak identifier, outbreak status) and outbreak details (e.g. outbreak setting, number of confirmed cases, severity indicators (hospitalizations and deaths)). These data are primarily collected by local public health units and summarized at the provincial and territorial level prior to submission to PHAC. Some optional variables included public health measures and population at risk, as available or feasible. Certain P/Ts were unable to participate, and other jurisdictions were unable to provide the information for some variables.

Each province and territory had their own SARS-CoV-2 outbreak definitions which were operational and often setting-specific (e.g. long-term care facility (LTCF) vs. workplace). Standardization across jurisdictions was not possible; therefore, CCOSS applied a standard definition to the outbreaks reported to the system, despite needing to exclude some from the database. The national outbreak definition was developed in consultation with the provinces and territories, to ensure consistency and comparability.

Two or more confirmed cases of COVID-19 epidemiologically linked to a specific setting and/or location. Excluding households, since household cases may not be declared or managed as an outbreak if the risk of transmission is contained. This definition also excludes cases that are geographically clustered (e.g., in a region, city, or town) but not epidemiologically linked, and cases attributed to community transmission (Public Health Agency of Canada, 2022a).

Data from CCOSS can be linked, with provincial and territorial permission, to the national COVID-19 case surveillance system. The case-based surveillance system captures data on demographics, clinical status and outcomes, exposures, risk factors, vaccination, and variant lineages for individual cases. This allows for automated data capture of outbreak-associated cases as well as reduced reporting burden by participating provinces and territories.

### Data storage and management

A second round of provincial and territorial consultations occurred in October–November 2020, followed by two cycles of federal and provincial and territorial approvals. In January 2021, all approvals were finalized, and provinces and territories began voluntarily submitting outbreak data on a weekly basis using secure online platforms. Provinces and territories were encouraged to submit all outbreaks and available data declared since January 2021 in their preferred format (i.e. line list, submission through Respiratory Outbreak Summaries on the Canadian Network for Public Health Intelligence (CNPHI)); exclusion criteria based on the national COVID-19 outbreak definition were applied upon submission to PHAC. Outbreak line lists were cleaned and mapped to the CCOSS structure to ensure standardized coding of outbreaks and variables; PHAC sought clarifications from provincial and territorial partners to ensure accurate mapping to CCOSS. Data were subsequently ingested into a robust Postgres database, capable of hosting large amounts of data on internal servers, and were accessed through a connected Metabase web platform. Data quality checks were conducted on a weekly basis prior to ingestion, and mapping of data elements was updated as required.

All data mapping, analysis, and reporting were done using R (R Core Team, 2022).

## Outcomes

### Process outcomes

Eight P/Ts have contributed data to CCOSS; these jurisdictions represent over 93% of the Canadian population (Statistics Canada, 2022). The large P/Ts all agreed to participate, along with several smaller jurisdictions, making CCOSS fairly representative of the Canadian population.

**Fig. 2:**
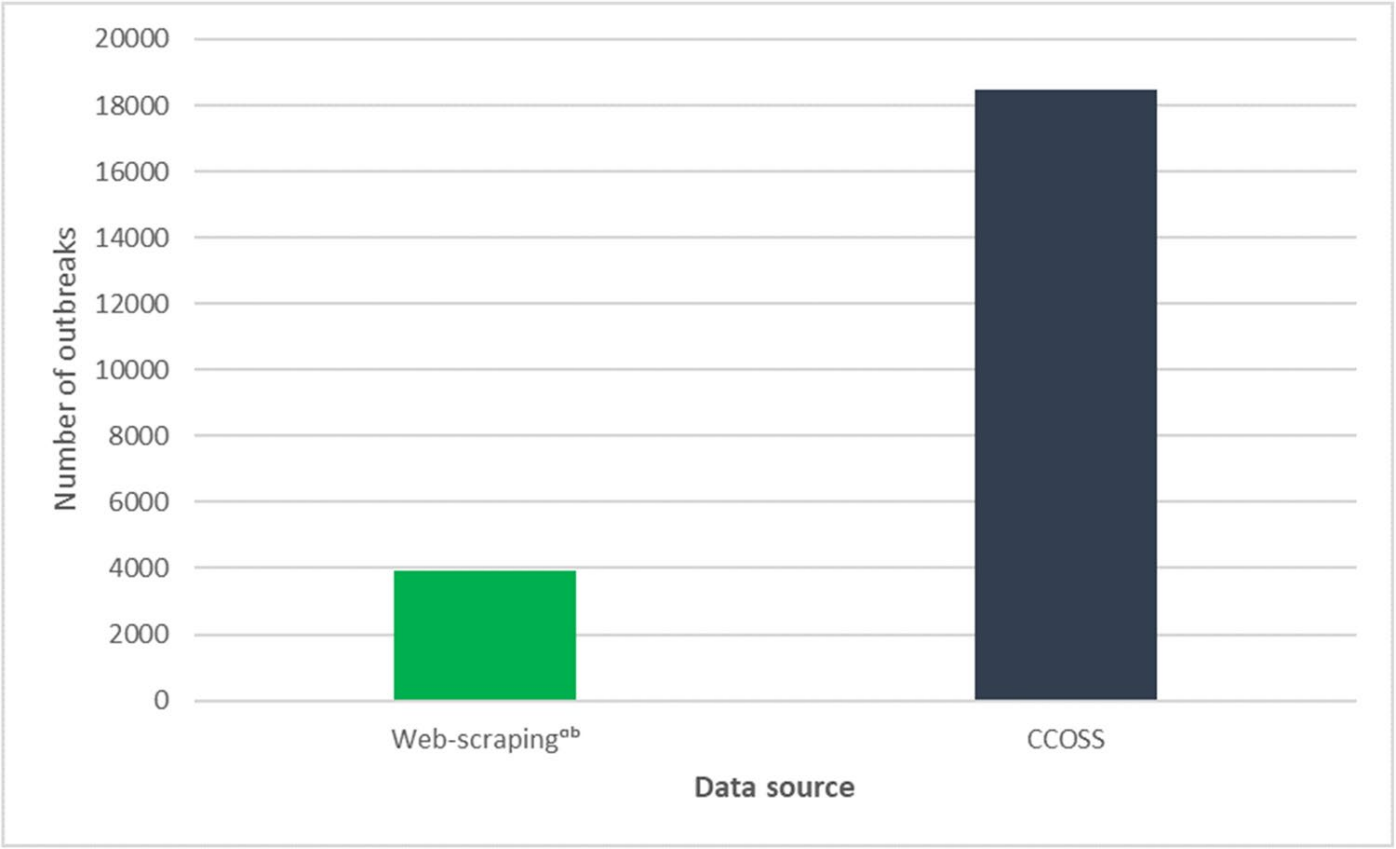
Number of COVID-19 outbreaks captured by web scraping vs. the Canadian COVID-19 Outbreak Surveillance System (CCOSS) from January to June 2021. ^a^May include duplicate outbreaks, ^b^includes Indigenous community outbreaks

Upon the implementation of CCOSS, PHAC conducted an analysis to examine how the interim outbreak surveillance system compared to data directly submitted from provinces and territories. This analysis illustrated that CCOSS captured substantially more outbreaks from January to June 2021 than the interim web-scraping system (Fig. 2). Following implementation of CCOSS in January 2021, the non-traditional outbreak surveillance system continued to run in parallel with a focus on provinces and territories that were not participating in CCOSS until January 2022 when it was discontinued.

### Public reporting

**Fig. 3:**
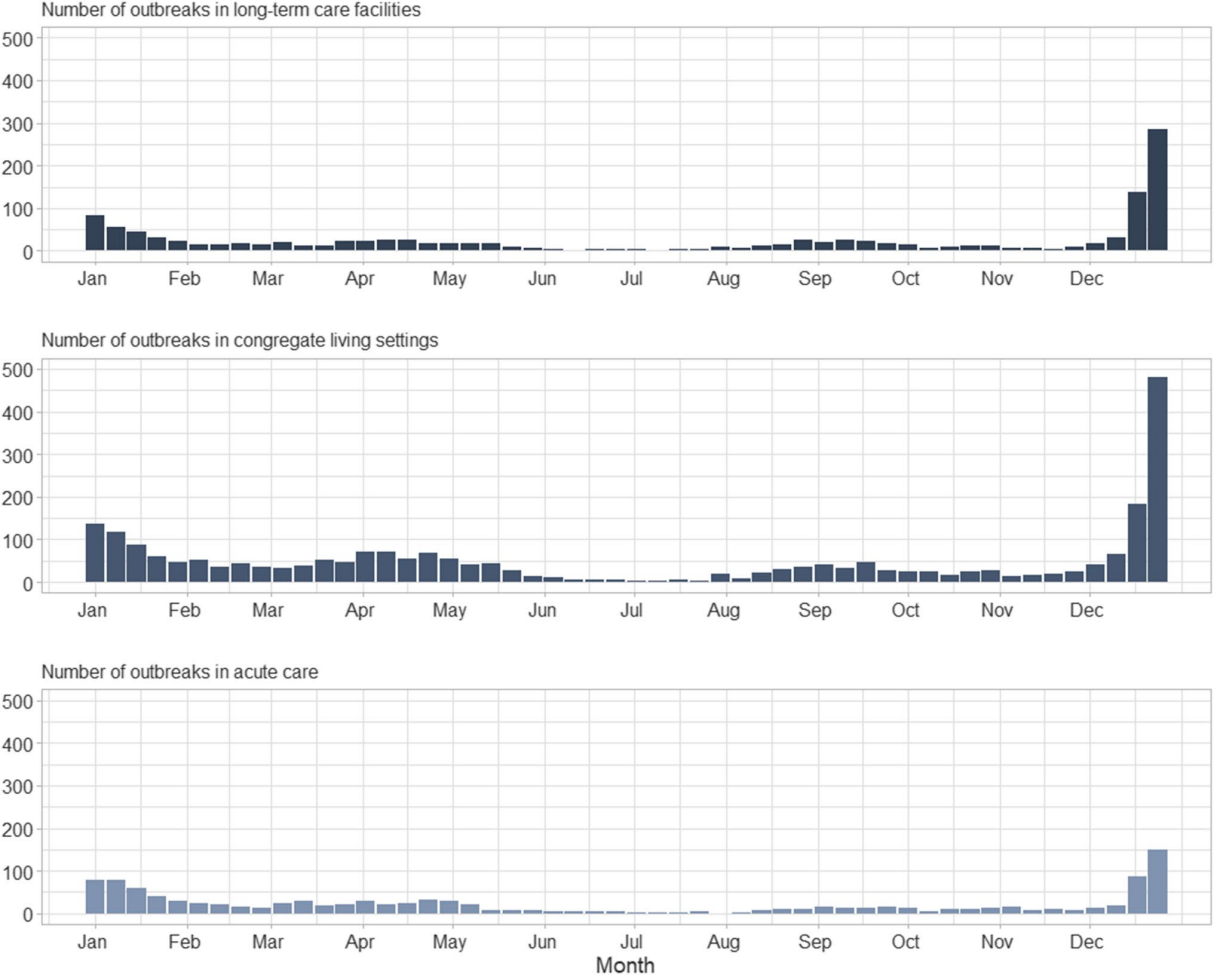
Weekly incidence of reported outbreaks in long-term care facilities, congregate living settings, and acute care by week from January to December 2021

**Fig. 4:**
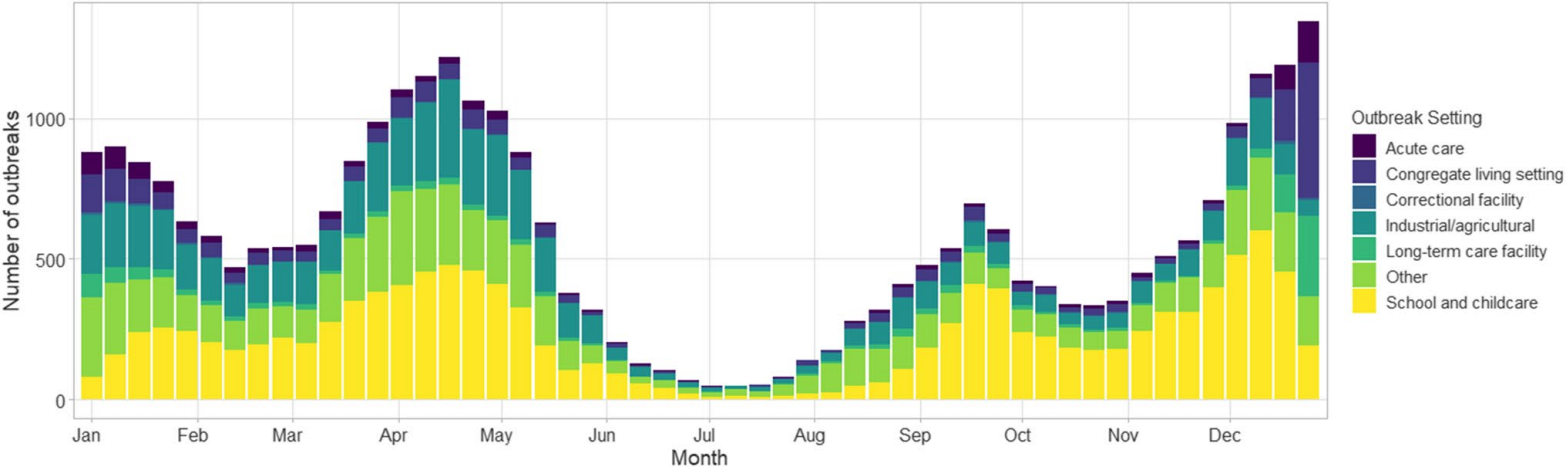
Weekly number of COVID-19 outbreaks by setting from January to December 2021

Data from CCOSS are available publicly through the Canada.ca website; outbreak incidence has been aggregated weekly by setting (Fig. 3). The public dissemination of outbreak trends permits users to examine nationally aggregated statistics (Fig. 4).

### Utility of CCOSS

Outbreak analyses have been useful to inform P/Ts in their effort to harmonize public health policy and response across jurisdictions and monitor the impact of public health measures (vaccinations, lockdowns, closures) on setting-specific outbreak incidence. Examples include timing for booster vaccines, immunization eligibility, and lockdowns. Evidence generated from CCOSS analyses has also been used to support several provincial and territorial outbreak investigations.

Beginning in 2020, a member of the PHAC COVID-19 Outbreak Response Unit (ORU) was paired with mobilized field epidemiologists to provide support and/or outbreak response tools (e.g. setting-specific questionnaires). In January 2021, with the launch of CCOSS, national data trends for a particular setting could be used to inform recommendations during field mobilizations. For example, how does the current school outbreak compare to school outbreaks on average (outbreak size, severity).

LTCF outbreak trends, including incidence of outbreaks, outbreak size, and severity outcomes, were monitored closely in fall 2021 to help inform recommendations on LTCF, including the administration of third COVID-19 vaccination doses for residents. School outbreak trends were contrasted with case incidence and jurisdictional school closures to examine correlations between closures and pediatric case incidence. Furthermore, outbreak trends (weekly incidence, outbreak size, and age distribution) were monitored following the vaccination rollouts for individuals aged 12–17 years and 5–11 years in various stages of school and childcare settings to assess the effect of vaccination on outbreaks.

### Challenges

The development and implementation of a novel Canadian COVID-19 outbreak surveillance system during a pandemic provided unique challenges. Eight of 13 Canadian provinces and territories participate in CCOSS. Despite geographical under-representation, participating jurisdictions represented a large proportion of the Canadian population (~ 93%), including both large P/Ts and smaller jurisdictions (Statistics Canada, 2022). CCOSS, like most surveillance systems, may underrepresent Indigenous communities, rural communities, and priority populations (Harper et al., 2011; Patterson et al., 2017; Souza et al., 2010; Zahnd et al., 2019; Zhang et al., 2017). Further, outbreak trends reported in a nationally aggregated manner may not be representative or align with individual jurisdictions’ outbreak trends.

Among provinces and territories submitting outbreak data to PHAC, some were unable to submit all proposed variables. Five out of eight provinces and territories indicated that outbreak control measures were not variables that would be routinely available. Further, legislation and policies within some provinces and territories prevented data sharing on more granular regional locations, ethnicity, and Indigenous status. In some provinces and territories, the roles and responsibilities of public health are shared with or under the jurisdiction of Indigenous health authorities. While CCOSS engaged Indigenous Services Canada (ISC) during the conceptualization of CCOSS, various barriers impacted participation. For example, ISC worked with Indigenous communities to develop outbreak definitions and surveillance that best met the needs of communities; the available data did not align with national COVID-19 outbreak definition and could not be incorporated into CCOSS. The lack of representation from Indigenous communities is a limitation for CCOSS. A formal evaluation of CCOSS is underway. One aspect of the evaluation will be assessing the variables requested against the ones required to meet the surveillance objectives and ways to incorporate data that are relevant to communities but that may not align with national data standards for surveillance.

During stakeholder engagement activities, the five provinces and territories that do not submit data to CCOSS had indicated their support for a national outbreak surveillance system but were not able to participate during 2021. There was considerable pressure on local public health to be able to track and report on outbreaks in a timely manner, which presented a challenge given that case/contact management and ensuring adherence to public health measures are also local public health responsibilities. Challenges balancing public health delivery and data collection may have created barriers to participating in CCOSS despite jurisdictional support for national outbreak surveillance. Variations in public health capacity within provinces and territories impact the ability to track outbreak trends. Outbreak surveillance is dependent on the identification of cases by the provincial and territorial surveillance system, i.e. individual cases being tested, reported to the public health system, and then linked to a common exposure setting/event and having that outbreak identified and reported. During periods of high case incidence, case and contact management is often strained and reduced, leading to the under-ascertainment of outbreaks or cases linked to an outbreak. For example, during the Omicron surge beginning in December 2021, provinces and territories shifted to prioritizing high-risk populations (e.g. LTCF, acute care, certain congregate living settings) for polymerase chain reaction (PCR) testing, meaning that cases and outbreaks in other community settings (e.g. workplaces, recreational facilities, schools) were substantially under-reported (Public Health Ontario, 2021; Yuan et al., 2022); as of August 2022, many P/Ts have stopped outbreak surveillance in non-priority settings. Due to limitations in outbreak detection, comparing trends over time may not be appropriate for some settings.

Differences in the way settings are defined and structured at the provincial and territorial level make it challenging to make comparisons across settings. Certain biases inherent to settings cannot be easily quantified in CCOSS (e.g. transient populations in shelters, hospitalizations in acute care, *healthy worker* effect (Chowdhury et al., 2017) in workplaces).

Initially, CCOSS analyses results were distributed through national tables to P/T representatives and on the national website (June 2021 to June 2022); however, the report was not easily accessible due to the format of the website. Since October 2022, weekly analyses and aggregate data have been publicly available on the Canada.ca website alongside the national case data report. Finally, there is a reporting lag of approximately 2 weeks between outbreak declaration and reporting to CCOSS; outbreaks are declared at the local level, reported to provincial and territorial public health, and then submitted to CCOSS. As such, CCOSS may not be timely enough to act as an early warning system.

## Implications

The Canadian COVID-19 Outbreak Surveillance System complemented the case-based surveillance system and allowed the description of epidemiological trends of SARS-CoV-2 outbreaks in Canada. Here, we provide recommendations to ongoing SARS-CoV-2 outbreak surveillance and for future response to emerging pathogens.

### Detect

- Establish and maintain strong relationships and communications with provincial and territorial partners. In the Canadian context, early and continuous engagement with provinces and territories (as key data holders and partners) was a critical step to ensuring the success of CCOSS and will be imperative moving forward as data availability and surveillance goals change in the context of COVID-19.
- Establish a national outbreak definition and get a clear understanding of how outbreaks are defined at the local and provincial and territorial levels.
- Establish timeline reporting requirements for select high-risk settings (i.e. with potential for signal detection).
- Establish linkages between multiple surveillance systems to link case, laboratory, and outbreak data. The ability to link various data sources together enhances public health’s ability to detect emerging threats (Colijn et al., 2022) and improves the scope and detail of future analyses.

### Understand

- Build highly skilled and qualified surveillance teams. It was critical that surveillance system staff members had knowledge of current data management and analysis technology to ensure that data from different local outbreak surveillance systems could be collated into one federated system.
- Maintain a curated list of individuals with surveillance, software, and data management skill sets to ensure that teams could be easily pulled together during a future public health crisis.
- Ensure the surveillance team includes experienced epidemiologists who can analyze and interpret outbreak data in a practical, relevant way for public health action.
- Include laboratory identifiers in the case data to permit the genomic data to be linked with the epidemiological data (Colijn et al., 2022). Linking laboratory and epidemiological data sources together can provide valuable public health intelligence on transmission dynamics and setting-specific risks (Colijn et al., 2022).
- Establish data usage guidelines and gaps in information to inform data analysis and additional data requests from partners.
- Determine which data elements are required to meet surveillance objectives and which data elements would be related to research goals.

### Act

- Collaborate with provinces and territories to identify generic data elements and data collection methods in preparation for emerging threats to enable timely and accurate implementation of an outbreak surveillance system for future epidemics or pandemics.
- Determine what analyses were required at local, provincial and territorial, and national levels of public health to support decision-making and formulate future plans to incorporate these needs into the surveillance system and reporting priorities.
- Develop minimum dataset standards for future emerging respiratory pathogens informed by the data elements collected by CCOSS.
- Ensure the minimum dataset required for national surveillance can be supplied by P/T surveillance. Discussions for additional data elements required to answer research questions should be secondary.
- Set clear SMART objectives based on a formal evaluation of CCOSS’s original objective(s) and processes (Centers for Disease Control and Prevention, n.d.).
- Explore additional methods of information dissemination to enhance awareness of national surveillance systems, reports and resources to P/T, and local public health partners and stakeholders.
- Implement web scraping as an interim solution to collect outbreak intelligence until a formal surveillance system with P/T reported data has been established.

## Conclusion

Ongoing SARS-CoV-2 outbreak surveillance provides necessary data to support public health action by identifying disproportionately affected populations and settings. More efforts are required to understand the burden of SARS-CoV-2 outbreaks for Indigenous and other priority populations. Work to collect data and conduct retrospective analyses can examine the utility of public health measures such as lockdowns, vaccine passports, and other non-pharmaceutical interventions on the number of SARS-CoV-2 outbreaks over time. Outbreak surveillance should be a priority for emerging threats.

## Implications for policy and practice

What are the innovations in this policy or program?

- Understanding outbreaks of a novel pathogen provides information on populations affected and improves understanding for allocating public health resources. The Canadian COVID-19 Outbreak Surveillance System provided data that were not captured through case-based surveillance. This system was novel due to the ability to produce trends on intensity, severity, and different settings with outbreaks as the unit of analysis.
- The frequency and integration of data submissions from many partners allowed rapid preparation of reports for public health officials.
- The system leveraged the pandemic emergency orders which allowed for timely information sharing from jurisdictions with greater detail than in pre-pandemic times.

What are the burning research questions for this innovation?

- Response planning for emerging and re-emerging infectious disease should include timely implementation of outbreak surveillance. Research is required to develop methods to capture outbreak trends for Indigenous populations and communities disproportionately impacted by social determinants of health.
- Partnerships to develop methods for data collection, and relevant indicators, should be prioritized in preparation for future health risks.
- As multiple public health risks are emerging (e.g. monkeypox, polio, ebola), research to integrate outbreak surveillance to better understand populations at risk and public health measures (e.g. masking, vaccination) could enhance our understanding of the utility of these measures in different settings and recommendations to respond to outbreaks.

## Data Availability

Available upon request to the corresponding author

## Footnotes

1 Cases associated with a specific setting or location and time with reasonable likelihood of transmission

## Code availability

Available upon request to the corresponding author.
